# Long-term normalization of calcineurin activity in model mice rescues Pin1 and attenuates Alzheimer’s phenotypes without blocking peripheral T cell IL-2 response

**DOI:** 10.1186/s13195-023-01323-5

**Published:** 2023-10-17

**Authors:** Nancy R. Stallings, Melissa A. O’Neal, Jie Hu, Zhong-Jian Shen, James S. Malter

**Affiliations:** Department of Pathology, University of Texas Southwestern Medical Center, 5323 Harry Hines, Dallas, TX 75390 USA

**Keywords:** Alzheimer’s disease, *APP/PS1* mice, FK506, Tacrolimus, Calcineurin, Pin1, Neuroinflammation

## Abstract

**Background:**

Current treatments for Alzheimer’s disease (AD) have largely failed to yield significant therapeutic benefits. Novel approaches are desperately needed to help address this immense public health issue. Data suggests that early intervention at the first stages of mild cognitive impairment may have a greater chance for success. The calcineurin (CN)-Pin1 signaling cascade can be selectively targeted with tacrolimus (FK506), a highly specific, FDA-approved CN inhibitor used safely for > 20 years in solid organ transplant recipients. AD prevalence was significantly reduced in solid organ recipients treated with FK506.

**Methods:**

Time release pellets were used to deliver constant FK506 dosage to *APP/PS1* mice without deleterious manipulation or handling. Immunofluorescence, histology, molecular biology, and behavior were used to evaluate changes in AD pathology.

**Results:**

FK506 can be safely and consistently delivered into juvenile *APP/PS1* mice via time-release pellets to levels roughly seen in transplant patients, leading to the normalization of CN activity and reduction or elimination of AD pathologies including synapse loss, neuroinflammation, and cognitive impairment. Pin1 activity and function were rescued despite the continuing presence of high levels of transgenic Aβ_42_. Indicators of neuroinflammation including Iba1 positivity and IL-6 production were also reduced to normal levels. Peripheral blood mononuclear cells (PBMC) obtained during treatment or splenocytes isolated at euthanasia activated normally after mitogens.

**Conclusions:**

Low-dose, constant FK506 can normalize CNS CN and Pin1 activity, suppress neuroinflammation, and attenuate AD-associated pathology without blocking peripheral IL-2 responses making repurposed FK506 a viable option for early, therapeutic intervention in AD.

**Supplementary Information:**

The online version contains supplementary material available at 10.1186/s13195-023-01323-5.

## Background

Alzheimer’s disease (AD) is one of the predominant health threats of an aging population in our modern, industrial society. With the recent exceptions of Aduhelm and Lecanemab, all drugs directed at Aβ_42_ have failed to gain FDA approval or show significant therapeutic efficacy [1, 2]. Failed trials were ascribed to heterogeneous patient populations with substantial co-morbidity but similar results have been observed in presymptomatic, homogeneous populations carrying dominant, hereditary risk factors including amyloid precursor protein or presenilin mutations [3]. These results have collectively suggested that either the therapeutic target or the timing of the intervention was wrong.

While Aβ_42_ and tau remain the predominant targets for the treatment of AD, another candidate gene is Pin1. Pin1 is the only known peptidyl-prolyl isomerase responsible for the *cis* to *trans* or *trans* to *cis* conversion of the peptide bond between a phosphorylated Ser or Thr and adjacent, C-terminal Pro [4]. Pin1-mediated isomerization alters target protein conformation, location, and function [4]. Pin1 is highly expressed in healthy brain but markedly reduced during AD development [5]. Brain regions that display early AD pathology including the hippocampus and entorhinal cortex show lower Pin1 expression than regions resistant to AD and lose expression as disease develops, suggesting cause and effect [6]. Mechanistically, Pin1 has been implicated in AD development through the regulation of amyloid precursor protein (APP) processing [7] and tau phosphorylation and conformation [8, 9]. Pin1 isomerizes APP at the peptide bond between threonine 668 and proline 669 facilitating APP processing through the non-amyloidogenic pathway [10]. Reductions in phosphorylation at threonine 668 or in Pin1 activity increase Aβ_42_ production [10, 11]. Pin1 also binds to phosphorylated tau, converting pathologic *cis*-tau to functional *trans*-tau [9, 12, 13]. Abolishing Pin1 activity through genetic knockout or pharmacologic blockade led to rapid synaptic loss in vitro and in vivo and subsequent neuronal cell death [7, 14]. We have shown that signaling induced by soluble, multimeric Aβ_42_ rapidly caused the inactivation of Pin1 and led to dendritic spine loss. The susceptibility to Aβ_42_ signaling provides a mechanism for how isomerase activity can be pathologically decreased very early in AD [14].

In a search for how Aβ_42_ signaling inhibited Pin1, IP/MS analysis of synaptoneurosomes followed by confirmatory co-IP studies revealed that Pin1 and calcineurin (CN) directly interacted [14]. CN is a very abundant, ubiquitously expressed, Ca2 + /calmodulin-dependent protein phosphatase which regulates a variety of cellular functions in multiple cell lineages including neurons, cardiac muscle, and lymphocytes [15–17]. Calcium dysregulation in neurons is a common feature in AD [18] which is initially driven by rising, soluble Aβ_42_ and causes CN expression and activity to increase [19]. We showed that CN inhibited Pin1 activity by dephosphorylation of Ser111 in response to Aβ_42_ [14]. These results suggested that CN inhibitors such as the FDA-approved immunosuppressants cyclosporin or tacrolimus (FK506) could reduce or prevent Aβ_42_ mediated pathologies such as synaptic loss and cell death associated with CN activation and Pin1 inhibition/loss. Indeed, FK506 preserved dendritic spines in wild-type but not Pin1 knockout neuronal cultures exposed to soluble, multimeric Aβ_42_ [14]. Additional lines of evidence also suggest FK506 is neuroprotective. Solid organ transplant recipients treated with FK506 had a significantly lower incidence of AD than similarly aged recipients immunosuppressed with other drugs or normal, age-matched controls [20]. Rapamycin prevents solid organ transplant rejection by blocking mTOR not CN and recipients of this drug showed no change in AD incidence [21]. Mechanistically, FK506 binds to CN and prevents substrate interactions and thus dephosphorylation of target proteins, including nuclear factors of activated T-cells (NFAT) and forkhead transcription factors (FOXO) [22]. NFAT dephosphorylation leads to activation of T-cells and IL-2 mRNA and protein production. Positive effects of FK506 include significantly reduced pilocarpine-induced epilepsy [23] and streptozotocin-induced memory impairment [24]. Short-term treatment with high dose FK506 promoted cognitive function in AD model mice [24, 25] but cognitive impairment and nephrotoxicity are common in overdosed transplant recipients [26]. The former likely reflects CNS accumulation of highly lipophilic FK506 in the brain [26–28] but suggests that doses inadequate for peripheral effects such as immunosuppression could be adequate to alter AD CNS pathology. Given the dearth of available therapeutics, the possibility of repurposing this FDA-approved drug, safely used for over 20 years in millions of patients, is worthy of consideration.

Here we have explored the feasibility and utility of long-term, continuous, moderate dose FK506 in *APP/PS1* mice [29]. In this model, plaque formation begins at ~ 1 month of age and rapidly increases thereafter [29]. Synaptic and neuronal loss, neuroinflammation, and behavioral deficits begin at approximately 6 months of age but tau pathology is absent [29]. Therefore, we initiated FK506 therapy at 3 months of age to model early disease and analyzed mice 3 months later for quantification of progression of AD pathologies. The drug was delivered subcutaneously for 90 days via time-release pellets that dissolve approximately 1%/day and are commonly used for long-term hormone therapy. We sought to stably maintain the mice at or slightly below the therapeutic level used in solid organ transplant recipients to minimize peripheral immune suppression and CNS side effects [30] and peak and trough variations. The data show that after an early spike, relatively stable FK506 plasma levels can be achieved through this approach and avoid daily handling, injections, or gavage of experimental animals. As in humans, FK506 accumulated in the brain and prevented or attenuated many AD pathologies including neuroinflammation, increased cytokine production, synaptic loss, and cognitive impairment and, importantly, did so without substantially affecting peripheral immune cell function as assessed by mitogen-induced, IL-2 mRNA expression from peripheral blood mononuclear cells (PBMC) harvested during therapy or splenocytes, harvested at euthanasia. These results are consistent with epidemiologic data from transplant recipients and suggest that the FDA-approved immunosuppressant FK506 could be effective in reducing AD pathology in humans.

## Results

### Continuous FK506 administration in APP/PS1 mice is feasible and safe without untoward effects

**Fig. 1:**
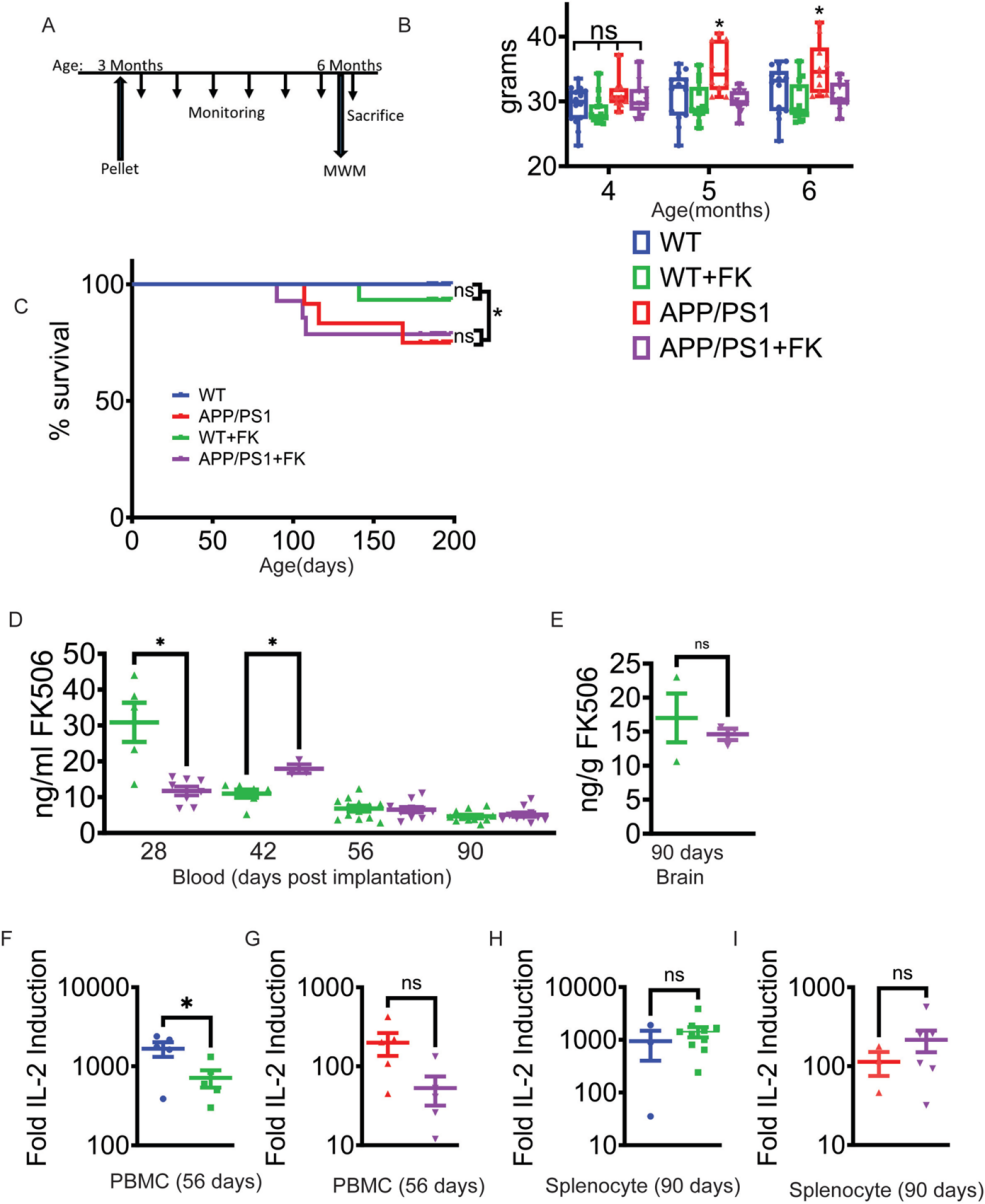
Long-term, continuous FK506 treatment in mice.** A**–**D** Experimental design, health status monitoring by weight, survival, and FK506 levels in WT and *APP/PS1* mice. **A** Experimental design indicating monitoring of weight, immune response, cognitive testing, survival, and FK506 levels. **B** Weight measurements of the mice during the duration of the experiment. Data are means ± SEM; *n* = 11–14 mice per group; * = *p* < 0.05 by two-way ANOVA with Tukey with the *APP/PS1*(red) mice being significantly different than the other 3 groups and no difference between the WT (blue), WT + FK506 (green), and *APP/PS1* + FK506 (purple) cohorts. **C** Kaplan-Meyer survival curve. *N* = 11–14 mice per group. WT (blue), WT + FK (green), *APP/PS1* (red), and *APP/PS1* + FK (purple). **D** FK506 concentration in tail vein blood by mass spectrometry in the WT + FK506 (green) and *APP/PS1* + FK506 (purple) cohorts. *n* = 4–12 mice per group. * = *p* < 0.05 by two-tailed unpaired *t*-test. **E** Post-mortem brain analysis of FK506 levels in treated mice. *n* = 6–10 mice per group. **F** Peripheral blood mononuclear cell (PBMC) immune activation assay using IL-2 transcript levels at 56 days post pellet implantation in WT mice. Fold induction versus unactivated cells. **G** Peripheral blood mononuclear cell immune activation assay using IL-2 transcript levels at 56 days post pellet implantation in *APP/PS1* mice. **H** Splenocytes from WT sacrificed mice were activated with PMA/ionophore for 4 h prior to measurement of IL-2 mRNA by qPCR. **I** Splenocytes from *APP/PS1* sacrificed mice were activated with PMA/ionophore for 4 h prior to measurement of IL-2 mRNA by qPCR. Fold induction versus unactivated cells. Data are means ± SEM; *n* = (3–10) biological replicates. * = *p* < 0.05 by one-way ANOVA with Tukey. For all graphs, WT (blue), WT + FK506 (green), *APP/PS1* (red), and *APP/PS1* + FK506 (purple)

We employed the *APP/PS1* AD mouse model due to its well-characterized development of amyloid plaques, synaptic and neuronal loss, and subsequent behavioral deficits [29, 31–33]. Time release pellets (Innovative Research of America) containing FK506 or no drug (placebo) were placed subcutaneously between the scapula in male 3-month-old *APP/PS1* and age-matched, littermate C57/Bl6 mice. ~ 1% of the pellet dissolves/day, continuously releasing approximately 1 mg/kg/day of FK506 into the animal. Prior analysis of the effects of FK506 in AD model mice employed gavage or IP injections [34, 35] requiring daily animal handling and stress or drug dissolved in drinking water or mixed with food. While convenient, these two latter delivery modes will generate high peak and low trough levels, potentially inducing toxicity or be subtherapeutic, respectively. After pellet implantation, mice were housed in a non-sterile conventional facility and assessed for adverse effects of FK506 treatment by monitoring weight, appearance, and mobility (Fig. 1A). Untreated *APP/PS1* mice weighed significantly more at 5–6 months of age than FK506-treated wild-type, treated *APP/PS1*, or control C57/Bl6 mice (Fig. 1B), suggesting FK506 prevented weight gain and obesity [36]. Activity and mobility were indistinguishable between all mice (not shown). At euthanasia at 6 months of age after 3 months of treatment, white adipose fat cell size was greater in untreated *APP/PS1* mice, likely accounting for their increased weight (Supplemental Fig. 1 A-B). Morphologic examination of kidney, liver, spleen, and adrenal was unremarkable between controls and treated groups (Supplemental Fig. 2 A-D) demonstrating that there was no gross pathology in any of the analyzed tissues due to long-term treatment with FK506. There were no statistically significant differences in survival between the treated and untreated mice of either genotype (Fig. 1C), consistent with the safety profile of FK506 in this dose range [30]. There was a reduced survival in the untreated *APP/PS1* versus WT mice, a previously reported phenotype [37].

Tail bleeds were performed bi-weekly for plasma FK506 measurements by mass spectroscopy starting at 28 days (Fig. 1D). Due to the constraints of our mouse protocol, a small subset (3–4 mice per cohort) was sampled at each time point to limit the number of times an individual mouse was bled. The small groups of tested mice likely account for the variation in blood FK506 levels seen on days 28 and 42 of treatment, as one animal was an outlier that significantly affected the average. After behavioral testing at 6 months of age, blood was collected from all mice at sacrifice for a final FK506 measurement (Fig. 1D). Brain lysates were also analyzed for steady-state FK506 levels by mass spectroscopy as done with plasma and splenocytes were harvested for ex vivo activation. Brain FK506 levels were significantly higher (~ threefold) than plasma (15 ng/g compared to 5 ng/ml) (Fig. 1D, E) demonstrating the concentration of the highly lipophilic FK506 in the CNS. Similar data has been observed in humans [38].

FK506 levels spiked initially but maintained steady-state levels of 4.5–6.5 ng/ml for the majority of the experimental period (Fig. 1D) which is at the trough level for solid organ transplant recipients (3–8 ng/ml) [30]. In order to establish if peripheral T cell immunocompromise occurred during treatment, peripheral blood mononuclear cells (PBMC, ~ 60% of total nucleated cells are T cells) were harvested after tail bleeds at 56 days of treatment and immediately activated for 4 h with ionophore and phorbol 12-myristate 13-acetate (PMA) prior to analysis of IL-2 mRNA by qPCR [39]; IL-2 is a CN- and NFAT-dependent cytokine essential for T-cell activation and proliferation [40, 41]. Data were compared to IL-2 mRNA levels in unstimulated cells of comparable genotype. In WT mice, PMA/ionophore activation increased IL-2 mRNA expression by ~ 700X in PBMC from FK506 treated, WT mice but ~ 1500-fold in PBMC from placebo-treated WT mice, a statistically significant but very modest biological suppression of IL-2 mRNA production. PBMC from untreated *APP/PS1* mice produced ~ 5–10X less IL-2 mRNA than untreated WT controls which was ~ 200-fold baseline. Despite trending lower, there was no significant difference in IL-2 mRNA production from treated *APP/PS1* PBMC after mitogens (Fig. 1G). These data suggest a modest blockade to activation in *APP/PS1* mice due to the transgenic mutations but that FK506 treatment had no independent, inhibitory effects or only modest ones. Splenocytes harvested immediately after sacrifice at 6 months of age and activated in vitro with the same PMA/ionophore protocol showed essentially identical data as observed in PBMC [39] (Fig. 1H, I) except for full recovery of IL-2 mRNA accumulation compared to genotype controls. These data suggest that the maintenance of FK506 below ~ 5–6 ng/ml, as measured at 90 days of treatment, fails to significantly suppress T cell activation from control. Second, we ascribe decreases in T cell IL-2 mRNA production in the AD model mice to a side effect of the *APP/PS1* transgenes. Therefore, FK506 was likely nontoxic and was without obvious adverse effects. Indeed, FK506 mice had significant improvements in weight and possibly other metabolic parameters.

### FK506 normalizes CN activity and increases the level of active, phosphorylated Pin1

**Fig. 2:**
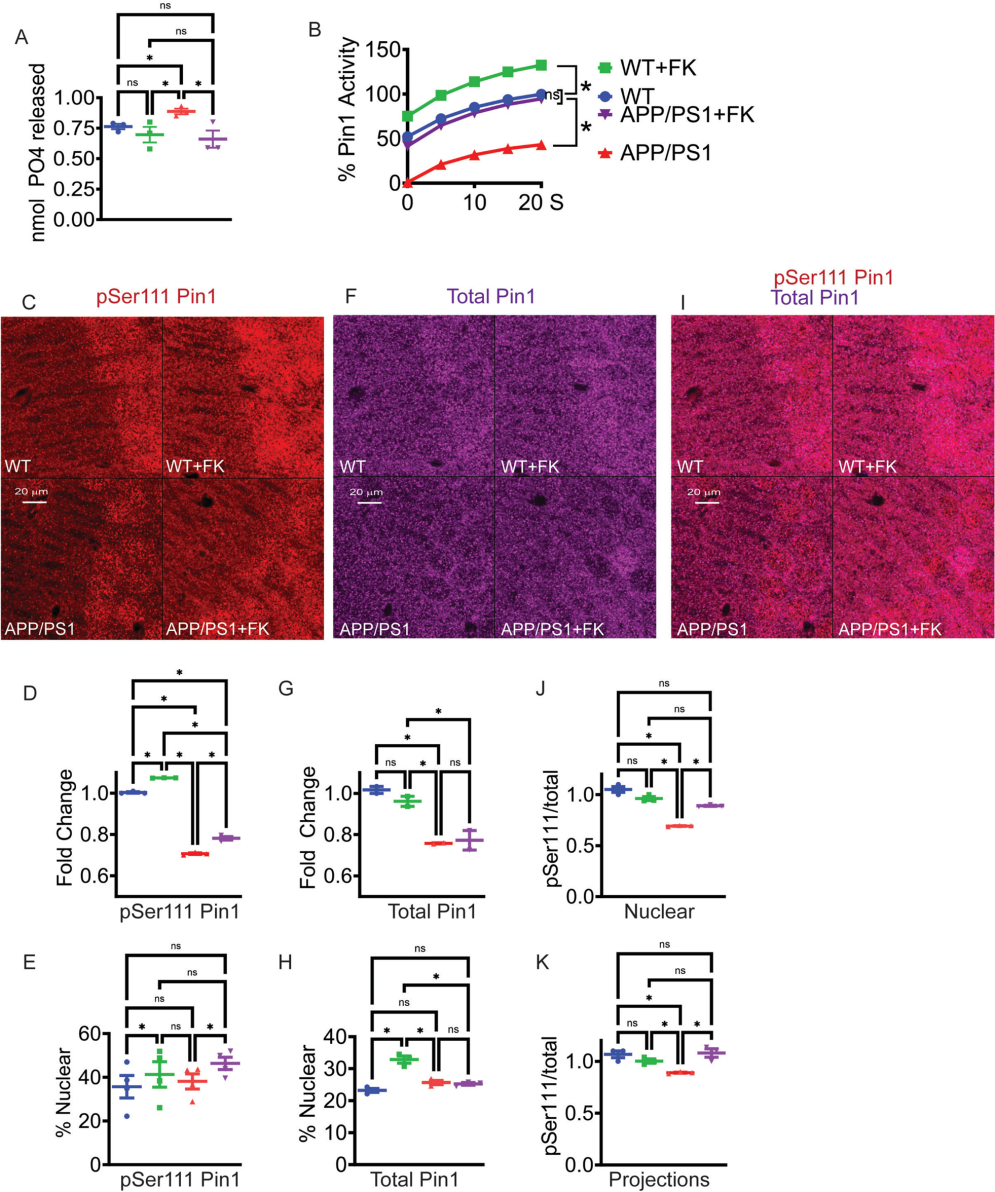
Pin1 and CN activity are normalized in FK506 treated mice.** A** CN activity assay. Data are means ± SEM; *n* = 3 biological replicates. * = *p* < 0.05 by one way ANOVA with Tukey. **B** Pin1 isomerase activity assay with brain lysates. Curves are normalized to WT activity 100%. n = 3 mice per group. * = *p* < 0.05 by a two-way ANOVA with Tukey. **C** Immunohistochemical analysis of pSer111 Pin1 in the hippocampus (CA1) of the experimental mice using anti-pSer111 antibody. **D** Quantification of the fold change of nuclear intensity for pSer111 Pin1. * = *p* < 0.05 by one-way ANOVA. **E** Quantification of percent of immunofluorescence present in the nucleus versus cell extensions for pSer111 Pin1. * = *p* < 0.05 by one-way ANOVA with Tukey. **F** Immunohistochemical analysis of total Pin1 in the hippocampus (CA1) of the experimental mice using total anti-Pin1 (rabbit) antibodies. **G** Quantification of the fold change of nuclear intensity for total Pin1. * = *p* < 0.05 by one-way ANOVA with Tukey. **H** Quantification of percent of immunofluorescence present in the nucleus versus cell extensions for total Pin1. * = *p* < 0.05 by one-way ANOVA with Tukey. **I** Double immunofluorescence with anti-pSer111 Pin1(rabbit) and total anti-Pin1 (mouse) in the CA1 of the experimental mice. **J** Ratio of pSer111 Pin1/total Pin1 in the nucleus of the CA1. * = *p* < 0.05 by one-way ANOVA with Tukey. **K** Ratio of pSer111 Pin1/total Pin1 in the apical projections of the CA1. * = *p* < 0.05 by one-way ANOVA with Tukey. Scale bar = 20 μm. For all graphs, WT (blue), WT + FK506 (green), *APP/PS1* (red), and *APP/PS1* + FK506 (purple)

These data clearly demonstrate that low-dose FK506 administered continuously, has very modest, if any effects on peripheral T cell activation. We previously reported that soluble, multimeric Aβ_42_ rapidly (seconds to minutes) inhibited Pin1 isomerase activity in synaptoneurosomes and cultured neurons, leading to loss of dendritic spines and eventual cell death [14]. Mechanistically, Aβ_42_ signaling activated CN which dephosphorylated Pin1 at Ser111, reducing isomerase activity [14]. As FK506 concentrates in the brain, we evaluated if CN activity, neuroinflammation, Pin1 activity, and other AD pathologies were attenuated by FK506 treatment despite ongoing Aβ_42_ production. To assess if these effects could be reversed in vivo, total cortical lysates were made from 6-month-old placebo or FK506-treated, wild-type, and *APP/PS1* mice and CN activity was first measured. As expected, lysates from untreated *APP/PS1* mice had significantly increased CN activity which was normalized to untreated WT levels in the *APP/PS1* FK506 treated mice (Fig. 2A). There were no differences in total CN immunoreactive protein based on western blot (Supplemental Fig. 3) suggesting FK506 reduced phosphatase activity without affecting CN gene expression or protein stability. *APP/PS1* placebo-treated mice had significantly reduced pan-cortical Pin1 isomerase activity compared to WT littermate controls (Fig. 2B). However, Pin1 activity was also rescued to WT levels in *APP/PS1* mice treated with FK506. These data suggest that peripherally delivered FK506 effectively crossed the blood–brain barrier and generated adequate CNS concentration to normalize cortical CN and Pin1 activities. Second, as hypothesized, FK506 treatment increased Pin1 isomerase activity in all animals including the WT cohort (Fig. 2B), suggesting the existence of a pool of inactive Pin1 present in mouse brain under normal, basal conditions. The effects of increased Pin1 activity in WT brains with FK506 treatment are unknown. Pin1 is highly overexpressed, often > fivefold in many malignant tumors, and facilitates cell cycle progression, making it a potential therapeutic target [42]. However, here FK506 treatment only elevated Pin1 activity by ~ 35%, and treated mice showed no untoward effects including metabolic derangements, behavioral abnormalities, gross tissue pathology, or tumor development during FK506 treatment.

CN regulates Pin1 activity via dephosphorylation of Ser111 [14], located 2 amino acids from the isomerase active site [43]. Additional phosphorylation events at Ser16 and 71 have also been implicated in the control of Pin1 activity [44, 45]. Therefore, we evaluated the impact of FK506 on total Pin1 and the degree of Ser111 phosphorylation in *APP/PS1* mice versus controls using pan-anti-Pin1 and anti-phospho-Ser111 antibodies (Fig. 2C–H). Consistent with Pin1 isomerase assay of cortical lysates shown above, there was a small but significant increase in Pin1 phosphorylated at Ser111 in WT mice treated with FK506 (Fig. 2C-D). FK506 treatment increased pSer111 staining in the CA1 of *APP/PS1* mice, although not to the levels seen in WT mice (Fig. 2C, D). There was a marked decrease in total Pin1 staining in the CA1 of the *APP/PS1* mice which was also not fully rescued by the FK506 treatment (Fig. 2F, G). However, the ratio of CA1 pSer111/total Pin1 in the nucleus and projections was the same in treated *APP/PS1* mice and the WT controls and significantly greater than seen in the *APP/PS1* untreated group (Fig. 2I–K). The early AD pathology of CA1 may reflect high levels of Aβ_42_ and elevated CN activity [46]. FK506 treatment also increased both total and pSer111 Pin1 nuclear staining in the CA1 of WT animals (Fig. 2E, H). Presumably Pin1’s nuclear localization augments neuronal transcriptional or DNA repair functions [47, 48] while increases in synaptic regions (Fig. 2K) account for spine protection [14]. Western blots of total Pin1 and pSer111 Pin1 from cortical lysates did not show any differences in amounts or pSer111 from any of the cohorts (Supplemental Fig. 4A-E) suggesting that there is regional and/or cell-specific difference in Pin1 responses to FK506. In aggregate, these data suggest that Pin1 amounts, phosphorylation, localization, and activity are regionally altered as *APP/PS1* mice age and AD pathologies spread cortically which can be partially reversed with FK506 dosages employed here.

### CN inhibition prevents spine loss and reduces amyloid plaque load

**Fig. 3:**
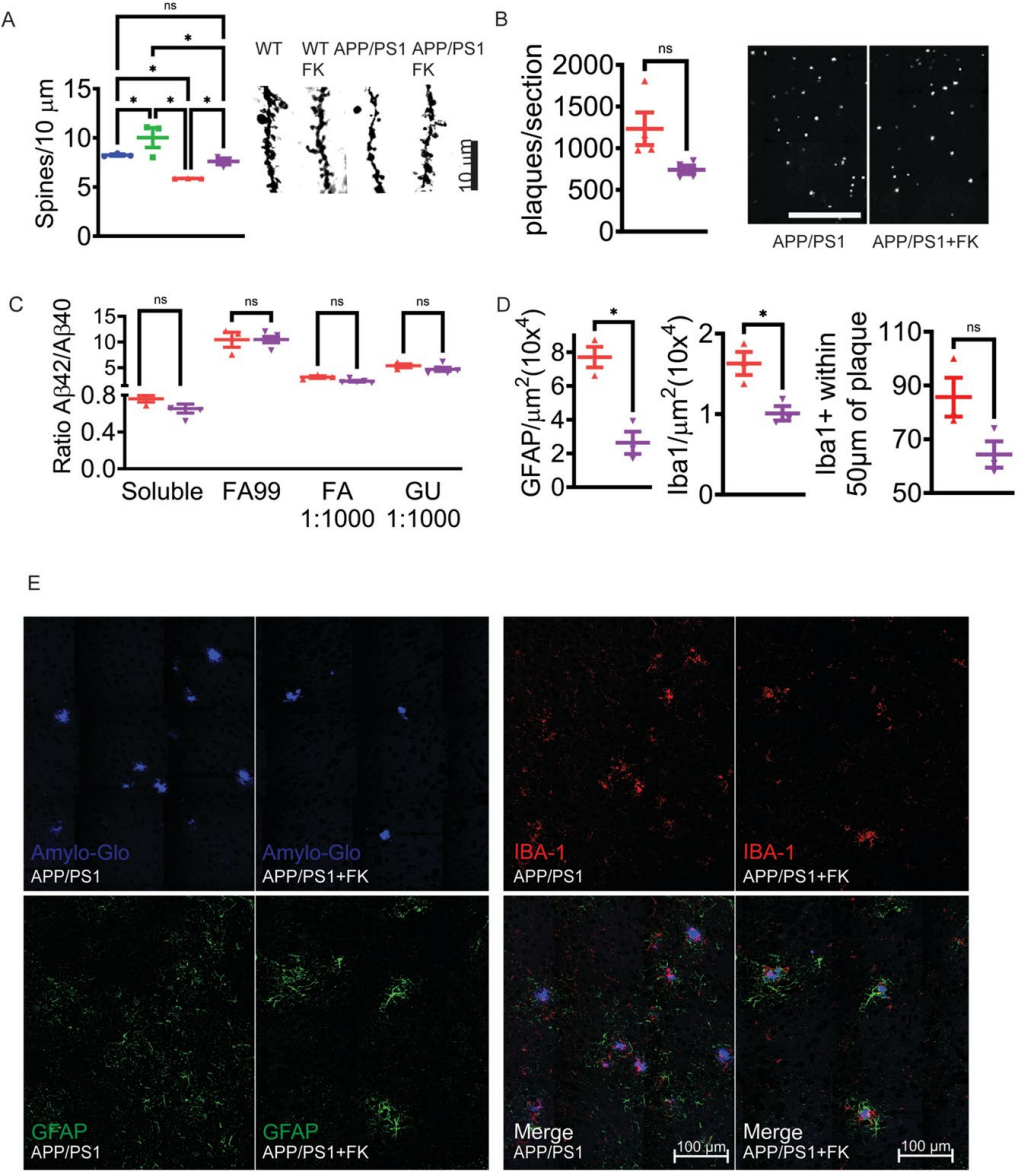
FK506 rescues spine counts and reduces neuroinflammation.** A** Cortical golgi staining for spine counts. *n* = 3 mice per group. * = *p* < 0.05 by a one-way ANOVA with Tukey. Scale bar = 10 μm. **B** Average numbers of amyloid plaques per cortical section of *APP/PS1* mice. *n* = 3 mice per group with 7–8 brain sections per mouse. * = *p* < 0.05 by a paired *t*-test. Scale bar = 500 μm. **C** β-amyloid 40 and 42 ratios in cortical lysates from *APP/PS1* mice. FA99 = formic acid 99% extraction. FA = 70% extraction. GU = glutaraldehyde extraction. *n* = 3–4 mice per group. * = *p* < 0.05 by a two-tailed unpaired *t*-test. **D** Number of GFAP immunoreactive cells and Iba1 immunoreactive cells per μm.^2^ and distance from amyloid plaques in the *APP/PS1* cortex. *n* = 3 biological replicates. For GFAP and Iba1 counts, 7–8 brain images were analyzed. For Iba1 distance from plaques, 25 plaques were analyzed per mouse and the number of Iba1 cells within 50 μm was counted. * = *p* < 0.05 by a two-tailed unpaired *t*-test. **F** Images used for quantification in **D**. Scale bar = 100 μm. For all graphs, WT (blue), WT + FK506 (green), *APP/PS1* (red), and *APP/PS1* + FK506 (purple)

Evolving AD is characterized by synaptic losses, brain inflammation, and accumulating plaque and tangle burden. As Pin1 has been implicated in dendritic spine maintenance [14], we counted spine numbers in treated versus control mice. As has been previously reported [49], we observed a significant reduction in spines in 6-month-old *APP/PS1* compared to placebo-treated WT mice which was restored to normal in *APP/PS1* mice treated with FK506 (Fig. 3A). We also saw a significant increase in the spine counts in FK506-treated WT mice over their placebo control (Fig. 3A), suggesting that low dose CN inhibitor can modulate spine number in healthy mice.

We next determined the number of amyloid plaques in the cortex of the treated and untreated *APP/PS1* mice using Amylo-glo [50]. We observed a significant reduction in the number of plaques in the cortex of the treated versus the placebo mice (Fig. 3B). Untreated *APP/PS1* mice produce 2500 to 12,000 pmol/g brain tissue of soluble Aβ_42_ [29]. Measurements of soluble and insoluble Aβ_40_ and Aβ_42_, as well as their ratio from the cortex of the mice did not show significant differences between the treated and untreated groups (Fig. 3C and Supplemental Fig. 4F, G). These data are important as they eliminate FK506-mediated reductions in Aβ_40_ and Aβ_42_ production or accumulation as driver for the observed mitigation of AD pathology in treated mice. However, these results suggest that the kinetics of plaque formation/remodeling are altered in the treated *APP/PS1* mice, possibly due to changes in the extracellular milieu that participates in plaque seeding or plaque turnover.

### FK506-treated mice show a reduction in neuroinflammation

CNS inflammation is a well-characterized and prominent feature of AD [51]. Aβ_42_ mediated microglial and astrocytic activation leading to free radical, chemokine, and cytokine release damages neurons and synaptic connections [52]. Pin1 regulates cytokine and chemokine production and release from T cells and eosinophils in the peripheral immune system [53, 54]. Therefore, we characterized the neuro-immunologic milieu of *APP/PS1* mice treated with FK506 by quantitating the number of GFAP-positive astrocytes and Iba1 + microglial cells in the cortex. Both showed significantly decreased numbers in FK506-treated *APP/PS1* mice (Fig. 3D, E), consistent with reduced inflammation and decreased neuronal damage in the brains of the treated mice. There was also a significantly decreased number of the remaining Iba1 + cells within 50 μm of the amyloid plaques in the treated *APP/PS1* mice. Activated microglia around amyloid plaques release toxic cytokines and oxidants, and attenuating this response should be beneficial in reducing neuronal damage [55].

**Fig. 4:**
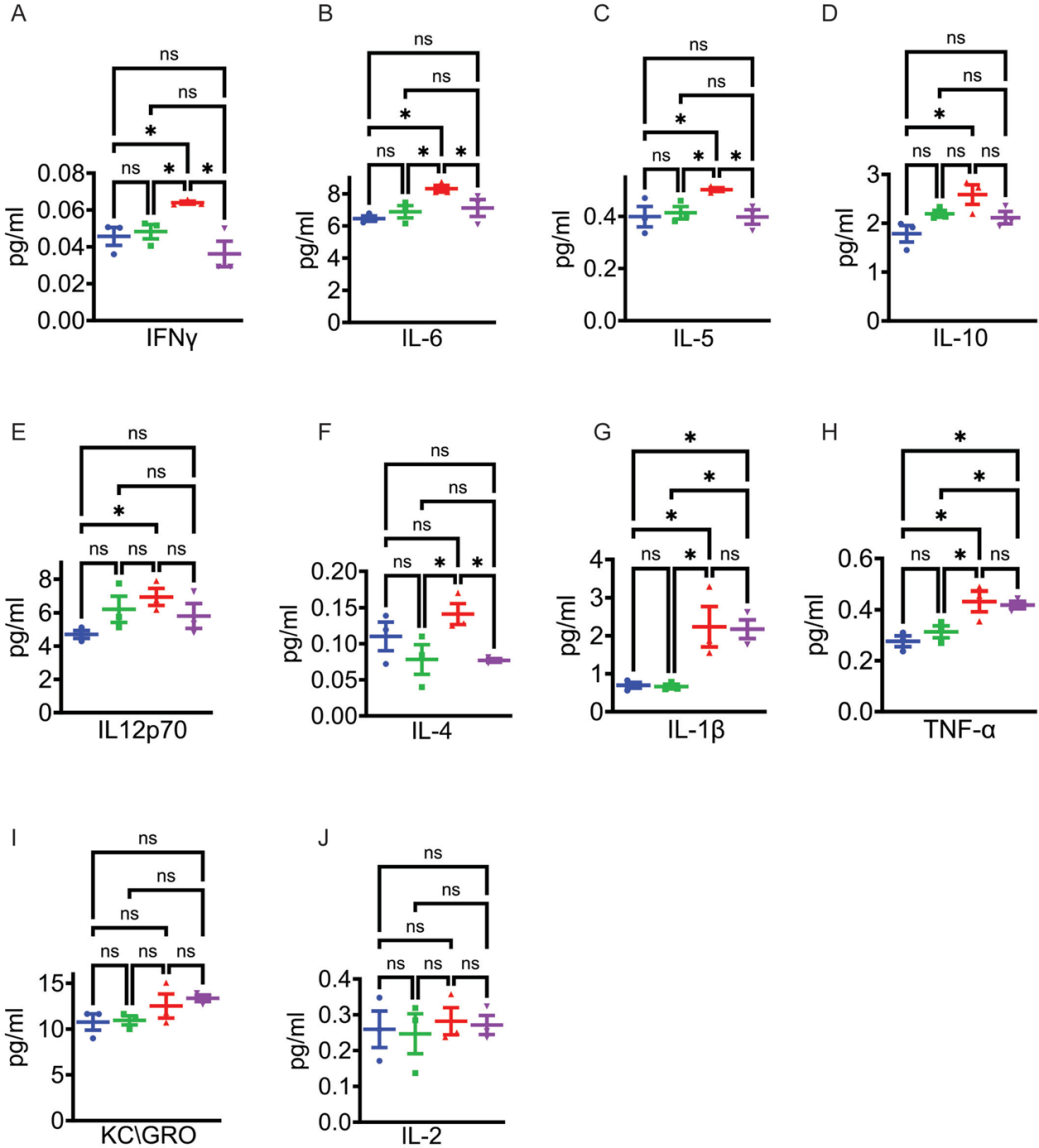
FK506 normalizes chronic cytokine production in the cortex of *APP/PS1* mice. Mesoscale multiplex analysis of cytokine levels in the cortex. **A** IFNγ, **B** IL-6,** C** IL-5, **D** IL-10, **E** IL12p70, **F** IL-4, **G** IL-1β, **H** TNF-α, **I** KC/GRO, **J** IL-2. *N* = 3 mice per group. * = *p* < 0.05 by one-way ANOVA with Tukey. For all graphs, WT (blue), WT + FK506 (green), *APP/PS1* (red), and *APP/PS1* + FK506 (purple)

The reduction in GFAP and Iba1 cells in the *APP/PS1* FK506-treated mice (Fig. 3D), suggested the cytokine expression would be reduced. As previously published, cortical levels of interferon gamma (IFNγ), IL-6, IL-5, IL-10, IL12p70, IL-1β, and TNF-α were all increased in the *APP/PS1* mice in comparison to WT mice (Fig. 4A–H) [56–58]. After 3 months of treatment IFNγ, IL-6, IL-5, IL-10, and IL12p70 were all reduced to the levels seen in the untreated WT mice (Fig. 4A–E). IL-1β and TNF-α, however, remained elevated and their levels unchanged with FK506 treatment (Fig. 3G, H). The levels of KC-GRO and IL-2 were the same in all cohorts (Fig. 4I, J). IL-4 was reduced in both WT and *APP/PS1* mice with the FK506 treatment (Fig. 4F). CN is a known transcriptional regulator of IL-4 [59]. Cumulatively, these data show that the FK506 treatment was effective in reducing much of the chronic neuroinflammation that is associated with AD phenotypes in the *APP/PS1* mice. In addition, the data raise the possibility that CN and Pin1 participate in microglial and astrocytic intracellular signaling which controls proinflammatory gene expression and responses to injury.

### FK506-treated mice show improved cognition

**Fig. 5:**
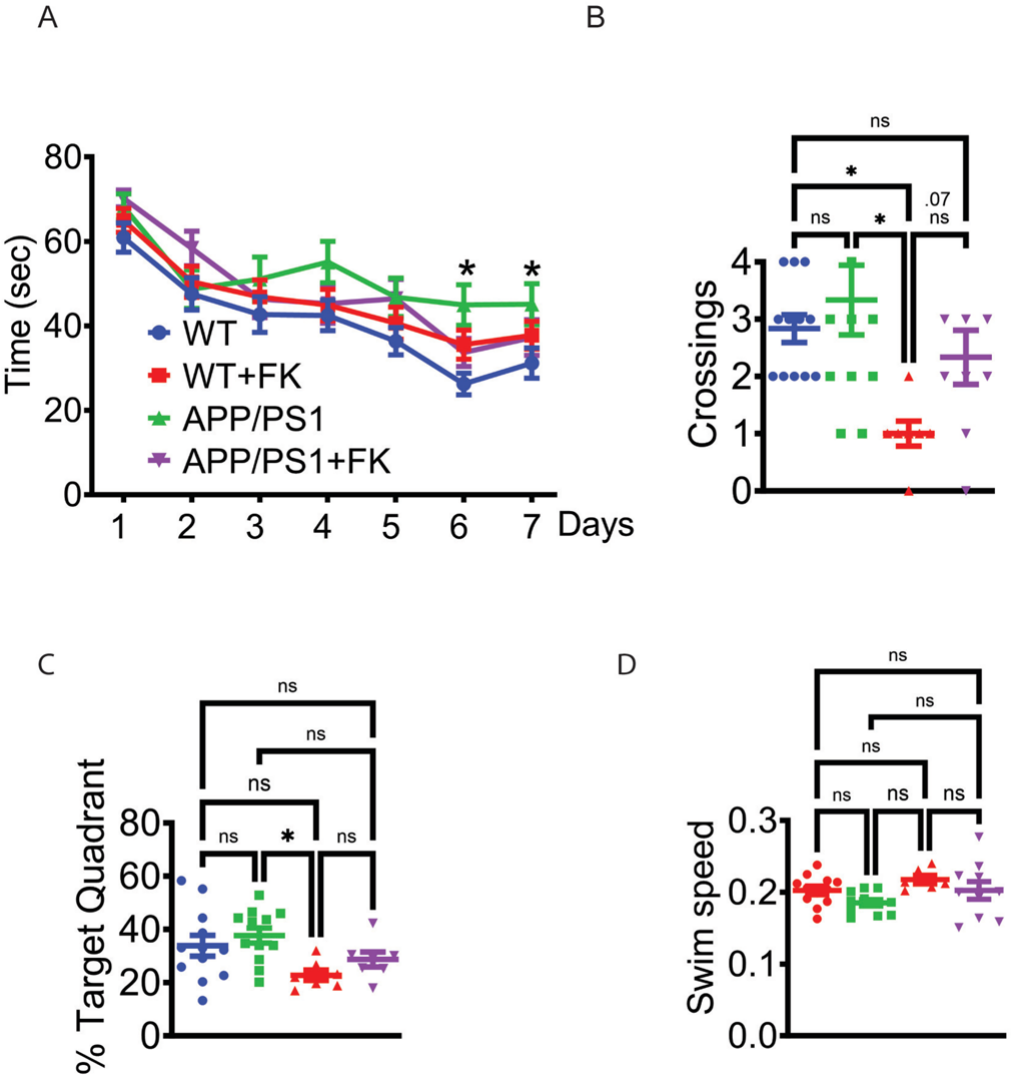
Treated *APP/PS1* mice show improved water maze performance.** A** Platform acquisition time during training from each of the four groups of mice. *n* = 7–12 per group, 4 trials per mouse per day. * = *p* < 0.05 by a two-way ANOVA with uncorrected Fisher’s LDS with a single pooled variance. Significance is between WT and *APP/PS1* mice at trial days 6 and 7. **B** Crossings from the probe trial for each of the four groups of mice. *n* = 7–12 per group. * = *p* < 0.05 by a one-way ANOVA with Tukey. **C** Percent time spent in correct Water Maze quadrant *n* = 7–12 per group. * = *p* < 0.05 by a one-way ANOVA with Tukey. **D** Swim speed in Water Maze. *n* = 7–12 per group. * = *p* < 0.05 by a one-way ANOVA with Tukey. For all graphs, WT (blue), WT + FK506 (green), *APP/PS1* (red), and *APP/PS1* + FK506 (purple)

Synaptic and neuronal loss during evolving AD is associated with cognitive impairment and memory deficits [60]. By 6 months of age, *APP/PS1* mice show significant reductions in Morris Water Maze (MWM) performance [29] consistent with spine losses and neuronal pathology. Therefore, we trained and tested control and treated mice and measured escape latency speed during the 7 days of training, crossings on the probe on the trial day (day 8), time spent in the target quadrant, and swim speed [61, 62]. A difference in escape latency was noted between the *APP/PS1* control and the other groups at training days 6 and 7 (Fig. 5A). Treated *APP/PS1* mice had normalized escape latency time compared to WT mice. In the probe trial, the *APP/PS1* FK506 mice crossed the target at the same rate as the WT mice, while untreated control *APP/PS1* mice did so at a significantly reduced rate (Fig. 5B). Time spent in the target quadrant mirrored escape latency (Fig. 5C). There were no significant differences in swim speed in any of the cohorts (Fig. 5D). These data suggest that preventing cognitive decline in FK506-treated mice despite the ongoing production of Aβ_42_ is possible if mice receive FK506.

## Discussion

Normalization of CN activity through the continuous, long-term delivery of FK506 significantly reduces the development and progression of multiple AD phenotypes in the *APP/PS1* mouse model. While acute CN blockade has shown protective effects in AD model mice, this study is the first to explore the efficacy of long-term, continuous FK506 at or below therapeutic levels used in human transplant recipients with the goal of normalizing CN and Pin1 activity in the brain. We have initiated therapy prior to overt pathology to mimic mild cognitive impairment (MCI) status, generated pharmacokinetic data over time, and determined if central and/or peripheral immune suppression occurs. Our studies notably revealed that Pin1 activity was normalized, amyloid plaque load was reduced, several metrics of neuroinflammation decreased, and dendritic spines were preserved in the hippocampus with significant improvement of cognitive performance on a commonly used behavioral test (MWM), all without blocking peripheral IL-2 response.

Two recently approved drugs for the treatment of AD (aducanumab and lecanemab) are anti-Aβ_42_ monoclonal antibodies that target amyloid plaques and soluble Aβ_42_ [63, 64]. Both drugs show modest benefits with significant side effects, require IV infusion, are very expensive, and in aggregate, are highly controversial [65, 66]. By contrast, FDA-approved FK506 is a small molecule that passes readily through the blood–brain barrier, is administered orally and available in generic formulations, has well-characterized mechanism of action and pharmacokinetics [67], and decades of safe clinical use [68]. Recent epidemiologic studies demonstrated that human transplant recipients treated with FK506 showed significantly lower AD incidence than normal, age-matched controls or other recipients treated with mTOR inhibitors [20]. Thus, we have proposed that the neuroprotective actions of FK506 could be achieved with a dose that is below the trough value of transplant patients with corresponding reductions in the risk of known side effects, particularly immunosuppression.

Several lines of evidence support using FK506 for AD treatment. Seven days of high dose FK506 improved fear-conditioning in Tg2576 mice, an AD model mouse which produces massive levels of Aβ_42_ [69]. Eight-month-old *APP/PS1* mice who received 5 mg/kg of FK506 delivered IP on alternate days for 60 days showed highly significant decreases in CN activity (~ 31%) with synaptic protein preservation, reduced neuroinflammation, and plaque burden [34]. However, the FK506 dosage was significantly higher than that used in transplant recipients and would likely be highly nephrotoxic as well as immunosuppressive in humans [34]. As such, it is very difficult to translate these initial proof-of-principal findings to feasibility in AD patients. Based on these provocative but incomplete mouse and human data, we asked if FK506, an FDA-approved compound with over 20 years of human use [70], could be safely repurposed at established therapeutic but ideally at even lower doses to treat early AD patients (so-called MCI patients), for whom effective therapy is modest. We also reasoned that continuous administration via time-release pellets rather than daily gavage or IP injection would also be advantageous to avoid peak concentration toxicity. Our data demonstrating rescue in dendritic spine counts, reduction of neuroinflammation, and normalization of MWM performance suggests not only an absence of neurologic side-effects of FK506 at the delivered doses but normalization of cognitive function.

These positive results are likely mediated, in part, through the preservation of Pin1 isomerase activity despite ongoing production and accumulation of pathological amounts of Aβ_42_. There are several lines of evidence that link Pin1 with AD pathology and normal plasticity. Pin1 expression is significantly reduced in AD patients as well as aging, AD model mice [6]. Pin1 redirects APP processing through non-amyloidogenic pathways [10] and prevents the pathogenic accumulation of *cis* hyperphosphorylated tau [8]. However, soluble concatemers of Aβ_42_ rapidly inhibit Pin1 function through the activation of CN, leading to synaptic loss, and eventually neuronal death [14]. Elevated ROS levels characteristic of AD oxidize Pin1 at active site Cys113 [71], also contributing to reduced isomerase activity. A presumed, de novo inactivating mutation of Pin1 was recently identified in AD patient neurons that correlated with elevated tau AT8 immuno-reactivity [72]. Normally Pin1 stabilizes the Kv4.2-DPP6 complex [73] and enhances NMDA-R function [74] both contributing to cognitive flexibility and plasticity. Despite its clear and essential role in normal neuronal function, global pharmacologic manipulation of Pin1 carries risk as a variety of malignant tumors overexpress Pin1 [75]. In addition, there are no established direct Pin1 activators. Thus, Pin1 enhancement strategies to combat AD must be localized to the brain and act upstream of Pin1 while leaving isomerase activity in other tissues at normal levels.

The identification of CN as an Aβ_42_-driven, Pin1 regulator created an opportunity to assess the potential of FK506 to preserve Pin1 in the brain, reduce inflammation, and prevent AD pathology. CN inhibitors have been employed previously as neuroprotectants and CNS immunoregulators. Cyclosporin-A, the first-generation CN inhibitor, has been proposed for the treatment of MCI [76]. In a spinal cord injury model, FK506 reduced neuronal inflammation and damage [35]. Overexpression of TDP-43 decreased CN expression [77], reducing plaque burden in an *APP/PSEN1* mouse model [77]. FK506 decreased mouse body weight despite a high-fat diet and reduced astrocytic activation in the ARC [78], effects we also observed here. FK506 at 1 mg/kg/day was protective in a mouse model of cerebral ischemia with suppression of microglial and astrocytic activation and peripheral immune responses [79]. Despite maintaining intact peripheral immunity, we observed significant reductions in Iba1- and GFAP-positive cells in the brains of treated mice. These data suggest that low levels of FK506 can have potent CNS effects without peripheral activity or toxicity. VIVIT, a CN inhibitory peptide, protected against the loss of synapse-related proteins and synaptic function in models of traumatic brain injury [80]. In total, there is mounting evidence that normalization of the CN pathway is beneficial for many neurodegenerative disorders. However, CN KO mice show severe behavioral and cognitive defects and FK506 overdose in transplant recipients causes neurotoxicity [81–84]. Based on the above and our data, there appears to be a reasonable therapeutic window for the safe and efficacious use of FK506 to potentially lessen AD progression without severe side effects. Further study to identify the lowest effective plasma concentration of FK506 to normalize CN activity is needed.

Based on the data presented here, it is likely that in addition to Ser111 phosphorylation, there are other post-translational modifications that are maintained after CN blockade important for retaining Pin1 isomerase activity. Despite complete rescue of cortical Pin1 isomerase activity in the *APP/PS1* FK506 treated mice, pS111 levels were not restored to WT levels. Several additional post-translational modification sites are known to regulate Pin1 activity including DAPK1-mediated Thr71 phosphorylation which is triggered by Aβ_42_ signaling [45, 85, 86]. Interestingly, DAPK1 is activated by CN and thus sensitive to FK506 [87]. DAPK1 is known to increase in TBI, resulting in increased pThr71 and hyperphosphorylated Tau [88]. It is reasonable to speculate that Pin1 regulation in the development of AD is a complex event with multiple phosphorylation and dephosphorylation events that control aggregate Pin1 isomerase activity.

Tau pathology is a critical component of AD [60]. However, only mice carrying mutant human tau transgenes produce neurofibrillary tangles. Tau repeat expressing cell lines show constitutively reduced Pin1 activity that was rescued with FK506 (Stallings et. al, unpublished data) suggesting these mouse models could also be sensitive to CN blockade. Consistent with this hypothesis, CN is elevated in tau overexpressing mice and inhibition of CN rescued their memory and spine defects [89]. It is likely that neuroinflammation driven by overexpression of Aβ_42_ leads to tau pathology which in turn leads to more neuroinflammation [90]. Therefore, FK506 has the potential to attenuate multiple neuronal insults leading to AD pathology.

Our results also demonstrate for the first time that FK506 can reduce the progression of AD phenotypes without profound peripheral immune suppression. The majority of plasma FK506 levels during the 90-day treatment hovered near trough levels observed in transplant patients. At these concentrations, we observed near normal activation kinetics of PBMC and splenocytes after mitogens. This somewhat unexpected result may reflect the absence of widely fluctuating daily levels (from peak to trough) due to continuous time release via the implanted pellets versus bolus clinical doses 12 h apart [30]. FK506 release from the pellet was greatest during the first few weeks after implantation. Similar kinetics have been observed for estrogen release from the pellet matrix [91]. Additionally, our monitoring of FK506 levels was influenced by the small sample size (*n* = 3–4) at each time point with high levels of FK506 in a single mouse able to significantly influence the mean. Despite these issues, we did not observe kidney and/or liver histopathology despite our long-term dosage schedule. High doses of FK506 in mice can decrease cognitive function [92] but at nontoxic levels can act as a regenerative agent [93]. A major unanswered question is how low can FK506 levels be with retention of protection against AD pathologies? Lower doses than used here will further reduce the risks of immunosuppression and toxicity but may not be neuroprotective. New technologies for implantable drug delivery systems have the potential to offer precise drug release and deliver drugs directly to target tissues [94]. For instance, nanoparticles that could effectively transport FK506 to the brain could be used to further reduce the risk of peripheral immune suppression [95]. It is also possible that a systemic lower daily dose would still be neuroprotective due to the lipophilic nature of FK506 and the accumulation of the drug in the brain.

Neuroinflammation is increasingly recognized as a driver of AD [96]. Temporary cytokine production and microglial activation may initially be beneficial in AD by clearing accumulating Aβ_42_, but chronic cytokine production and microglial activation is neurotoxic [97]. Microglia activation and tau propagation increase proportionately and progressively over Braak stages, consistent with neuroinflammation as a driver of neurofibrillary tangles and neuronal loss [98]. Thus, modulating cytokine levels and microglia activity is likely beneficial. We showed that FK506 was able to decrease many CNS proinflammatory cytokines and reduce the numbers of active glia, reducing neuroinflammation and increasing neuronal health. Based on our and other’s preclinical data [99], we propose that FK506 is a promising FDA-approved drug for early intervention in MCI. The critical issues to be defined remain how much FK506 should be used, when it should be given to patients and what are the most appropriate biomarkers to monitor its efficacy and side effects.

### Limitations

The present study includes early-stage experiments with long-term dosage of FK506 in AD model mice. AD model mice do not replicate human disease completely. Furthermore, small sample sets where *n* = 3–4 per treatment groups can limit significance. However, we view this as proof of concept demonstrating the viability and safety of long-term administration of FK506. 3R principles were used for the minimal number of mice to obtain statistically significant data.

## Conclusions

FK506 is a candidate drug to be repurposed for the treatment of Alzheimer’s disease. This small molecule is inexpensive, has well-established pharmacokinetics, crosses the blood brain barrier, and accumulates in the brain. Our novel data builds upon the FK506 literature in neurological disorders by showing reduction in neurodegeneration and neuroinflammation through the normalization of CN and Pin1 activity. Mice had no significant peripheral changes in IL-2 mRNA expression at the dosage of FK506 used in this study suggesting that it is possible to identify a dosage of FK506 that is neuroprotective but not immune suppressive. In summary, our data suggests that FK506 should be evaluated as a treatment for AD.

## Materials and methods

### Mice

*APP/PS1* [29] mice were maintained on a C57/Bl6 background by crossing pure C57/Bl6 mice with heterozygous *APP/PS1* mice. Mice were fed a standard chow (Teklad Global 16% Rodent Diet, #2016) ad libitum and were housed in a standard 12-h light/dark cycle. Male mice were used for all experiments. Mice were monitored for health status by observation and weight. Weight measurement data are means ± SEM; *n* = 11–14 mice per group; * = *p* < 0.05 by two-way ANOVA with Tukey post-hoc test. Survival was analyzed on a Kaplan–Meier survival curve with a log-rank (Mantel-Cox) test. *n* = 11–14 mice per group. Upon sacrifice, half of mice were perfused with 4% PFA and half were processed as unfixed tissue. All mouse work was done in accordance with protocols approved by The University of Texas Southwestern Medical Center Institutional Use and Animal Care Committee.

### FK506 pellets

2.25 mg FK506/90-day, time release pellets were obtained from Innovative Research of America (Sarasota, Fl) (calculated to deliver 1 mg/kg/day per manufacturer). Pellets were implanted subcutaneously in the subscapular region of the mouse. Placebo pellets with same carrier material but lacking FK506 were used as control pellets. Mice were monitored for any adverse health events of the FK506 pellets by weekly weight measurements and observation. Any deaths of mice in the experiment were recorded.

### FK506 measurements

For measurement of FK506 in peripheral blood, blood was collected from the tail vein into an EDTA Microvette (Sarstedt). 20 μl of blood was spotted on a Whatman 903 Protein Saver Card. Concentrations of FK506 were then determined by the UT Southwestern Preclinical Pharmacology Core using standard methods to determine FK506 concentrations [100, 101]. Each individual mouse was bled no more than twice before sacrifice in accordance with our mouse protocol. For blood measurements, *n* = 4–12 per group. Significance * = *p* < 0.05 by two-tailed *t*-test with Holm-Šídák method. Brain tissue was provided to the UT Southwestern Preclinical Pharmacology core for analysis of FK506 levels. Blood measurements were used to correct for vasculature contributions of FK506 to the brain values. For post-mortem brain measurements,* n* = 6–10 per group. There was no significant difference between groups by two-tailed *t*-test with Holm-Šídák method.

### Peripheral blood mononuclear cell (PBMC) and splenocyte activation

To collect PBMC, blood from tail vein was collected in an EDTA Microvett tube (Sarstedt). Samples were incubated with 7 mL ACK lysis buffer at RT for 10 min with occasional vortexing to lyse red blood cells. Samples were washed twice in 7 mL PBS and spun at 1200 RPM for 5 min at RT. Pellet was suspended in 50 ul RPMI-1640 medium with 10% FBS and plated on a 96-well plate.

Freshly harvested spleens were pushed through a 40-µm cell strainer. Excess PBS completed spleen disruption and washed the strainer. Cells were centrifuged for 5 min at 1200 RPM at RT. Pellets were suspended in 5 mL ACK lysis buffer for 10 min at RT on a rocker. The samples were diluted with 20 mL PBS and spun as before. Samples were suspended in 25-mL PBS, passed through a second 40-µm strainer and spun as before. Samples were suspended in 5 mL RPMI-1640 medium with 10% FBS, diluted to 2 million cells/mL, and 1 mL was plated in a 24 well plate.

To activate PBMC or splenocytes, 16.2 nM phorbol 12-myristate 13-acetate (PMA) and 1 uM Ionomycin was added for 4–6 h. RNA was harvested with an RNAeasy micro kit (Qiagen) and treated with Turbo DNAse (ThermoFisher). cDNA was made with iScript (BioRad) and analyzed by qPCR. Relative gene expression was calculated with the 2^^−(ΔΔCT)^ method using IL-2 Forward5′-ATCAGCAATATCAGAGTAACTGTTGT-3′, reverse 5′TGTGTTGTCAGAGCCCTTTAGTTT-3′ and 18S primers forward 5′-ATCAACTTTCGATGGTAGTCG-3′, reverse 5′-TCCTTGGATGTGGTAGCG G-3′. Data are means ± SEM; *n* = (3–10) biological replicates. * = *p* < 0.05 by one-way ANOVA with Tukey.

### Mouse brain samples and western blots

Hemibrain minus cerebellum were flash frozen in liquid nitrogen upon sacrifice. Soluble fraction from mice hemibrain samples were prepared according to the method of Shankar et al. [102, 103]. 35 μg of brain lysate was run on a Stain Free Any kD™ gel (Bio-Rad, 456–9034), activated for stain free total protein, transferred to nitrocellulose, scanned on a Bio-Rad Chemidoc system, blocked with Li-Cor protein free blocking buffer, probed with primary antibodies and fluorescent secondary antibodies, and scanned using a Li-Cor Odyssey and analyzed using Image Studio Lite. The stain free gel membrane image was imported into Image Studio Lite and used to normalize for protein loading. Pin1 total antibody (mouse, Santa Cruz, sc-46660, 1:1000), Pin1 pSer111 (rabbit, Malter Lab, 1:1000), PPP3CB (rabbit, Invitrogen, pA5-15,581, 1:1000). Secondary antibodies donkey anti-rabbit Dylight 800 (Invitrogen, SA5-10,044, 1:10,000), donkey anti-mouse Dylight 680 (Invitrogen, SA5-10,170, 1:10,000). For western blots, WT values are set to 1 for normalization across multiple experiments. Data are means ± SEM; *n* = 3 biological replicates. * = *p* < 0.05 by one-way ANOVA with Tukey.

### CN activity assay

Tissue lysates were made from frozen mouse hemibrain slices on the same day as assay. Calcineurin Cellular Activity Assay Kit Colorimetric (Millipore, 207,007) were performed per manufacturer’s protocol. Plates were read at A_620_ on a Tecan plate reader Spark 10. Data is presented as nmol of PO4 released. Data are means ± SEM; *n* = 3 biological replicates. * = by one-way ANOVA with Tukey.

### Pin1 isomerase activity assay

Activity assays were performed essentially as described [14]. 1 μg of soluble fraction cortical brain lysate was used to assess Pin1 isomerase activity. Data was exported to Graphpad Prism® v9 for graphing and analysis. All isomerase assays were run with a BSA only control to assess spontaneous isomerization of the substrate. The BSA values were subtracted from the test samples using baseline subtraction. For graphing purposes, the samples were then normalized so that the BSA was set to zero and the WT C57/Bl6 samples were set to 100%. *n* = 3 mice per group. * = *p* < 0.05 by a two-way ANOVA with Tukey.

### Immuno-fluorescence

Immunofluorescence was done on 5-μm paraffin embedded sections deparaffinized by using a Tissue-Tek slide Stainer in the deparaffinization mode and then antigen retrieved using citrate buffer pH 6.0 (BioGenex, San Ramon, CA) in a microwave oven. Slides were washed 3 × in dH20 and then transferred to 1X TBS. Slides were blocked in 1X TBS, 0.3% triton, and 5% donkey serum. Primary and secondary antibodies were diluted in 1X TBS, 1%BSA, and 0.3% triton. DAPI (Invitrogen) was used to counterstain nuclei. Slides were mounted using Prolong Antifade Diamond (Invitrogen) or Aqua Polymount (Polysciences). Confocal Z-stack images were collected on a Zeiss LSM880 and 0.5 μm optical slices were obtained. Confocal acquisition settings were consistent for image quantification. Pin1 total antibody (mouse, Santa Cruz, sc-46660, 1:100), Pin1 pSer111 (rabbit, Malter Lab, 1:1000), Alexa Fluor 647 donkey anti-mouse (Invitrogen, A31571, 1:1000), Alexa Fluor 568 donkey anti-rabbit (Invitrogen, A10042, 1:1000).

### Nuclear intensity quantification

A macro was created in ImageJ to use the DAPI signal to measure fluorescence intensity in the nuclei. In a slice-wise manner, the DAPI signal was used to draw an outline of the nucleus for unbiased measurement of the other fluorescent channels present in the image. The signal intensity of the remaining channels was then measured within the DAPI ROI in a slice-wise manner to co-localize DAPI staining with other channels. Mean fluorescent intensity for each nuclei is obtained. Twelve 0.5-μm slices were used per image. Ten nuclei were counted per image and 3 images were analyzed and their intensities were averaged. For a set of images (WT, WT + FK506, *APP/PS1*, *APP/PS1* + FK506), all sections were on the same slide. For a set of images, fluorescent signal in untreated wild-type mice were set to 1 for normalization purposes across multiple sets of images. Macro available upon request. * = *p* < 0.05 by one-way ANOVA with Tukey.

### Nuclear percentage of fluorescence

A macro was made in Image J to create a mask from the DAPI/nuclear signal to separate nuclear signals from the projections. The macro segments the image into nuclei and cell projections in order to quantitate fluorescent intensity in all channels. The image was opened and the macro found the brightest slice to adjust brightness and contrast to avoid overexposure of the stack. A Z projection was created and max intensity was applied. A threshold was then applied to segment the nuclei from the projections. The macro calculates the percentage of signal that is in the nucleus versus projections. Macro available upon request. For a set of images (WT, WT + FK506, *APP/PS1*, *APP/PS1* + FK506), all sections were on the same slide. * = *p* < 0.05 by one-way ANOVA with Tukey.

### Golgi staining

The brain was dissected from the euthanized mouse and cut along the mid-line. Hemi brains were then processed according to the RapidGolgi kit (FD Neuroscience, Columbia, Maryland). Brains were sectioned and processed by FD Neuroscience for the Golgi stain. Bright field images of the apical projections from the CA1 were acquired on a Zeiss LSM 880 using a tiled Z-stack and a 0.2-µm thickness and analyzed using Neurolucida 360 (MBF Bioscience, Williston, Vermont). At least three independent fields were analyzed for each mouse. The experimenter was blinded to the genotype and treatment status of the mice during the counting. *n* = 3 mice per group. * = *p* < 0.05 by a one-way ANOVA with Tukey.

### Tissue preparation for Amylo-Glo, H&E, and immunofluorescence

Mice were deeply anesthetized prior to transcardial infusion with TBS and 4% PFA. Tissue was post-fixed in 4% PFA overnight before transfer to PBS for paraffin embedding. Tissue was processed and paraffin-embedded using standard methods by the UT Southwestern Molecular Pathology Core. Paraffin brain sections were 5 μm thick. Slides were prepared with a WT, WT + FK, *APP/PS1*, and *APP/PS1* + FK section on each slide. Brain sections were matched by morphology.

### Amylo-Glo

5-μm paraffin sections were cut and mounted on Superfrost Plus slides by the UT Southwestern Molecular Pathology Core. Deparaffinization was done using a Tissue-Tek Slide Stainer in the deparaffinization mode and then antigen was retrieved using citrate buffer pH 6.0 (BioGenex, San Ramon, CA) in a microwave oven. Slides were washed 3 × in dH20 and then transferred to 1X TBS. Sections were stained for Amylo-Glo (Biosensis) per the manufacturer’s directions. Briefly, sections were placed in 1X Amylo-Glo solution for 10 min, then rinsed in 0.9% saline for 5 min, and rinsed in distilled water for 15 s. Slides were then transferred into blocking buffer for immunohistochemistry. Blocking buffer was 5% goat serum, 5% donkey serum, and 0.2% Triton X-100. Primary antibodies used were anti-GFAP (1:1000, Biosensis, C-1373–50) and anti-Iba1 (1:250, Proteintech, 10,904–1-AP). Alexafluor secondary antibodies (A11039, chicken anti-goat 488 and A10042, donkey anti-Rabbit) were used at 1:1000. To-Pro-3 at 1uM (Thermo, T3605) was used to label nuclei per manufacturer’s protocol directions. Whole brain images were collected on a Zeiss Axioscan Z1.

### β-amyloid plaque counts

Axioscan files were imported into ImageJ using the BioFormats import tool. Channels were split and only the Blue Channel (Amylo-Glo) was retained. The adjust threshold tool was used to adjust the threshold for each image. Since brain sections (*APP/PS1* and *APP/PS1* + FK506) were matched on a slide, the same threshold was applied to each pair. The analyzed particles tools were then used to count the number of plaques per brain section using the settings of size 0-Infinity. The experimenter was blinded to the genotype/treatment of the mice during analysis. *n* = 4 mice per group with 7–8 brain sections per mouse. Sets of *APP/PS1* and *APP/PS1* + FK506 littermates were used for the comparison. * = *p* < 0.05 by a two-tailed paired *t*-test.

### GFAP and Iba1 quantification

Images were taken using a Zeiss LSM 880 confocal microscope. Tile scanning was used to encompass a larger area for analysis. 3 tile images per section were taken. At least 3 levels/sections were imaged. Only images where an amyloid plaque was present were analyzed. Image J was used to quantitate the distance of Iba1 immuno-reactive cells from the center of a plaque. First, the size and intensity of the plaque(s) were measured. Then, the distance from each Iba1 positive cell was measured. On the same images, the number of GFAP cells was also counted for number per image. *n* = 3 biological replicates. For GFAP and Iba1 counts, 7–8 brain images were analyzed per mouse. For Iba1 distance from plaques, 25 plaques were analyzed per mouse for the number of Iba1 positive cells within 50 μm of the plaque. * = *p* < 0.05 by a two-tailed unpaired *t*-test.

### *Aβ*_*40*_*, Aβ*_*42*_*,*  and cytokine measurements

Hemi-brains from *APP/PS1* mice were homogenized with 10 stokes with the loose pestle and 10 strokes with the tight pestle with 10% Wt/Vol of TBS + complete protease inhibitor. Homogenates were then spun for 1 h at 21000 g at 4C. The supernatant was collected and labeled TBS soluble fraction. The pellet was re-suspended in 99% or 70% formic acid or glutaraldehyde to the original homogenization volume, sonicated for 35 s, and spun at 21000 g for 1 h. The FA soluble fraction was equilibrated 1:20 in neutralization buffer (1 M tris base, 0.5 M Na2HPO4, 0.05% NaN3). Measurements were performed using the Mesoscale V-PLEX AB Peptide Panel 1 (6E10) Kit (K15200E) and V-PLEX Proinflammatory Panel 1 Mouse Kit (K15048D). For V-PLEX AB Peptide panel, *n* = 3–4 mice per group. * = *p* < 0.05 by a two-tailed unpaired *t*-test. For V-Plex Proinflammatory Panel, *n* = 3 mice per group. * = *p* < 0.05 by one-way ANOVA with Tukey.

### Morris water maze

The Morris water maze was conducted using a standard protocol [104]. A 120-cm-diameter pool was used that was filled with water and non-toxic opaque white paint. A platform of 10 cm was placed in the northeast quadrant of the pool one cm under the surface. A pseudo-random order was used to start the mice in varying quadrants. The mouse was allowed 75 s to find the platform before being guided by the experimenter to the platform. After either finding the platform or being guided there, the mouse was allowed to stay on the platform for 5 s before being removed. Four trials were conducted per day. Mice were trained for 7 days. The probe trial was conducted on day 8. The platform was removed, and the mice swam for 60 s. Time spent in each quadrant was recorded. Mice were recorded and analyzed by the HVS Water software (HVS Image). The experimenter was blinded to the genotype and treatment status of the mice during the duration of the behavioral assessment. For probe trial data, *n* = 7–12 mice. * = *p* < 0.05 using a mixed model two-way ANOVA with uncorrected Fisher’s LDS with a single pooled variance. For probe trial, percent time in quadrant, and swim speed,* n* = 7–12 mice. * = *p* < 0.05 by a one-way ANOVA with Tukey.

### Supplementary Information

**Additional file 1: Supplemental Figure 1. **White Adipose Tissue analysis. (A) H&E of white adipose tissue from WT, WT+FK506, APP/PS1, and APP/PS1+FK506 mice. Scale bar = 250 μm. (B) Quantification of adipose size from (A). *n*=3 mice per group. >400 adipose cells per mouse. * = *p* < 0.05 by one-way ANOVA with Tukey. For all graphs, WT (blue), WT+FK506 (green), APP/PS1 (red), and APP/PS1+FK506 (purple).

**Additional file 2: Supplemental Figure 2. **H&E of kidney, liver, spleen and adrenal. (A) H&E of kidney from WT, WT+FK506, APP/PS1, and APP/PS1+FK506 mice. Scale bar = 250 μm. (B) H&E of liver from WT, WT+FK506, APP/PS1, and APP/PS1+FK506 mice. Scale bar = 250 μm. (C) H&E of spleen from WT, WT+FK506, APP/PS1, and APP/PS1+FK506 mice. Scale bar = 250 μm. (D) H&E of adrenal from WT, WT+FK506, APP/PS1, and APP/PS1+FK506 mice. Scale bar = 250 μm.

**Additional file 3: Supplemental Figure 3. **CN levels in the cortex. (A) Immunoblot of PPP3CB from cortical lysates. (B) Fold change in PPP3CB levels compared to WT mice. n=3 mice per group. * = *p* < 0.05 by one-way ANOVA with Tukey. (C) Total protein loaded on blots from a Bio-Rad stain free gel and used for quantification in (B). For all graphs, WT (blue), WT+FK506 (green), APP/PS1 (red), and APP/PS1+FK506 (purple).

**Additional file 4: Supplemental Figure 4. **Cortical Pin1 expression and phosphorylation and Aβ40/42 levels. (A) Western blot of Pin1 from cortical lysates using anti-total and anti-pSer111 Pin1 antibodies. (B) Quantification of total Pin1 protein levels from (A) *n*=3 mice per group. (C) Quantification of pSer111 Pin1 protein levels from (A). *n*=3 mice per group. (D) Ratio of fold change of pS111/total Pin1 in Western blots. *n*=3 mice per group. (E) Total protein loaded on blots from a Bio-Rad stain free gel and used for quantification in (B-D). Mesoscale multiplex measurements of Aβ40 and Aβ42. (F) Aβ40 measurements from cortical lysates. *n*=3 mice per group. (G) Aβ42 measurements from cortical lysates. *n*=3 mice per group. (H) Images from Fig. 2 with secondary only controls.  All images adjusted to same intensity. For all graphs, WT (blue), WT+FK506 (green), APP/PS1 (red), and APP/PS1+FK506 (purple).

## Data Availability

Requests for data and material should be directed to James Malter (james.malter@utsouthwestern.edu). All unique material will be provided upon request.
