# Supplementary figures and images for: Long-term normalization of calcineurin activity in model mice rescues Pin1 and attenuates Alzheimer’s phenotypes without blocking peripheral T cell IL-2 response
Source: Alzheimers Res Ther. 2023 Oct 17;15:179. doi: 10.1186/s13195-023-01323-5 (PMC10580561; doi:10.1186/s13195-023-01323-5)

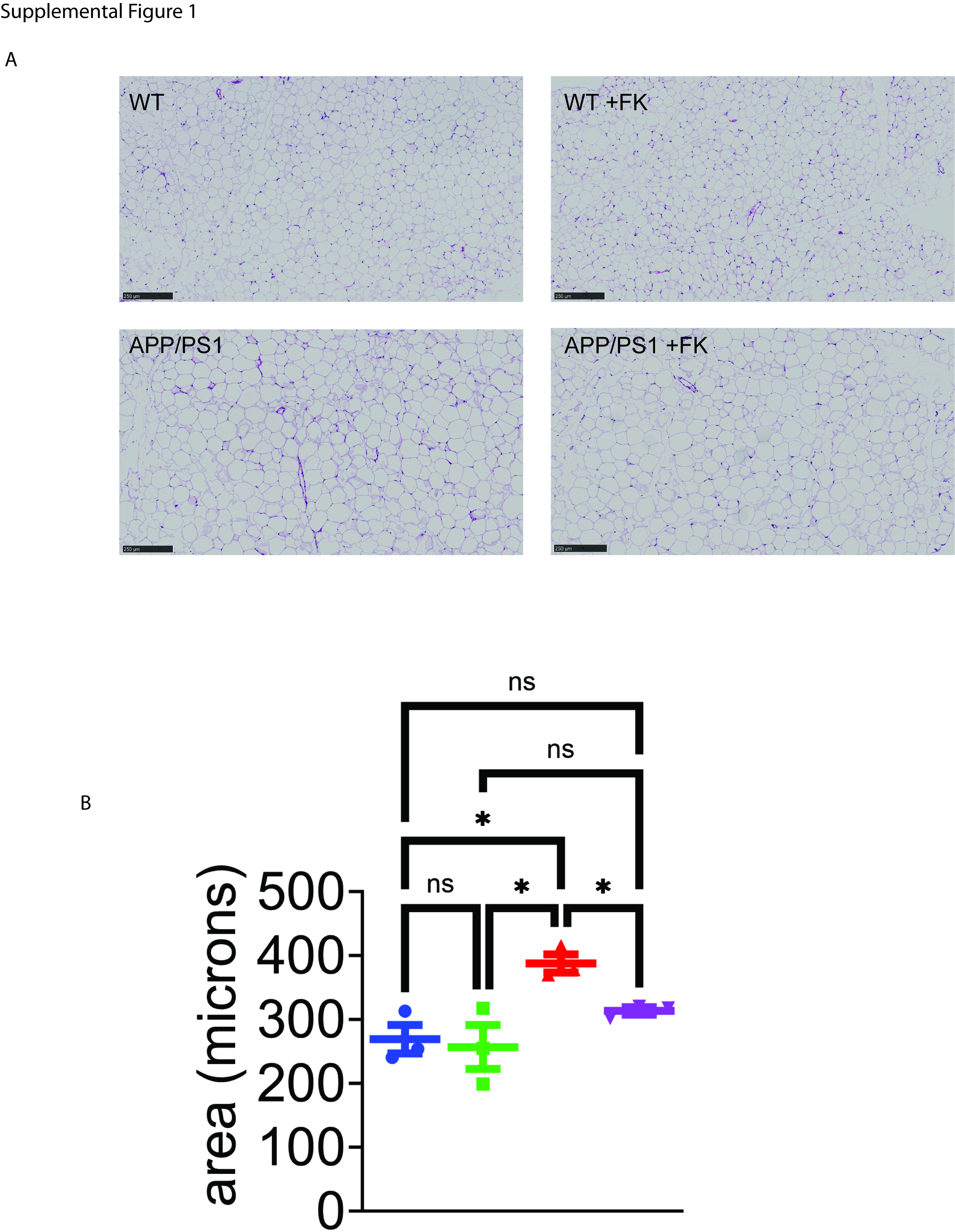

Supplement: Supplementary file 1 — Additional file 1: Supplemental Figure 1. White Adipose Tissue analysis. (A) H&E of white adipose tissue from WT, WT+FK506, APP/PS1, and APP/PS1+FK506 mice. Scale bar = 250 μm. (B) Quantification of adipose size from (A). n=3 mice per group. >400 adipose cells per mouse. * = p < 0.05 by one-way ANOVA with Tukey. For all graphs, WT (blue), WT+FK506 (green), APP/PS1 (red), and APP/PS1+FK506 (purple). [file 13195_2023_1323_MOESM1_ESM.tif]

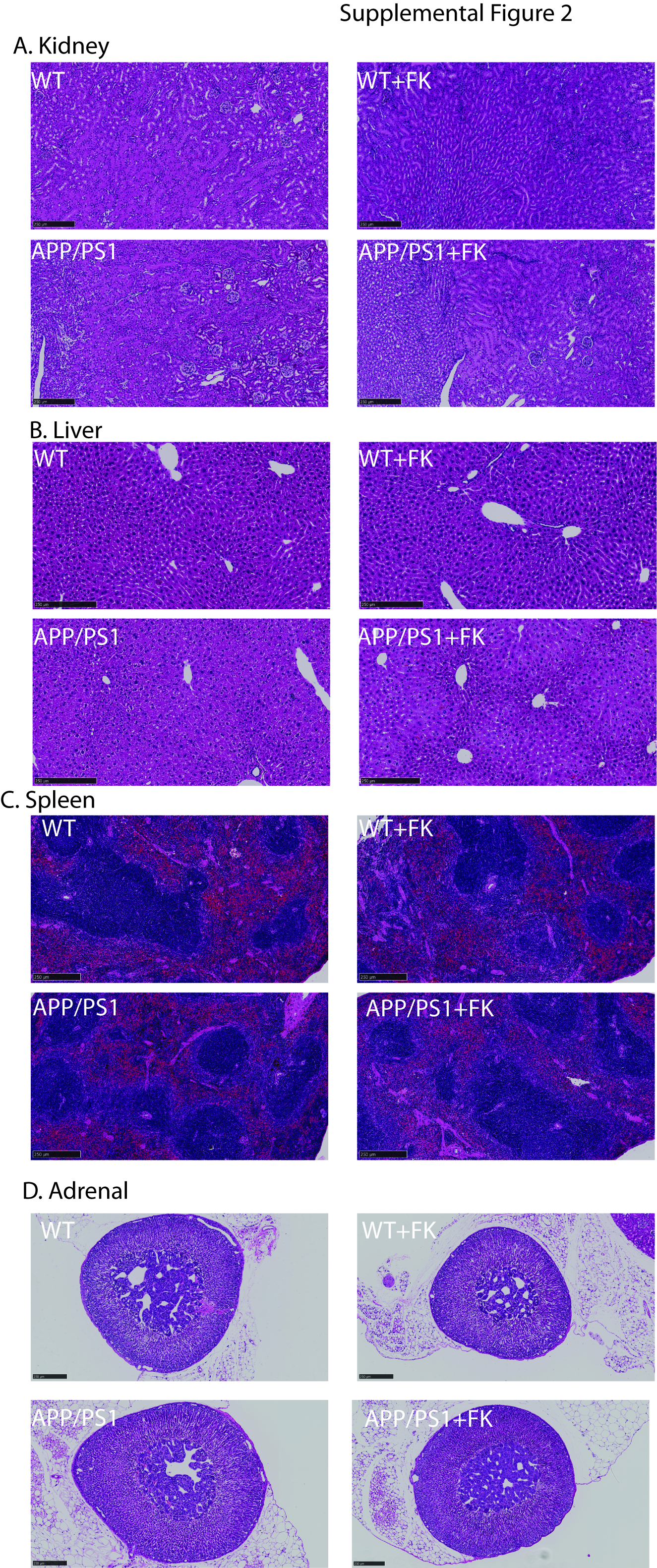

Supplement: Supplementary file 2 — Additional file 2: Supplemental Figure 2. H&E of kidney, liver, spleen and adrenal. (A) H&E of kidney from WT, WT+FK506, APP/PS1, and APP/PS1+FK506 mice. Scale bar = 250 μm. (B) H&E of liver from WT, WT+FK506, APP/PS1, and APP/PS1+FK506 mice. Scale bar = 250 μm. (C) H&E of spleen from WT, WT+FK506, APP/PS1, and APP/PS1+FK506 mice. Scale bar = 250 μm. (D) H&E of adrenal from WT, WT+FK506, APP/PS1, and APP/PS1+FK506 mice. Scale bar = 250 μm. [file 13195_2023_1323_MOESM2_ESM.tif]

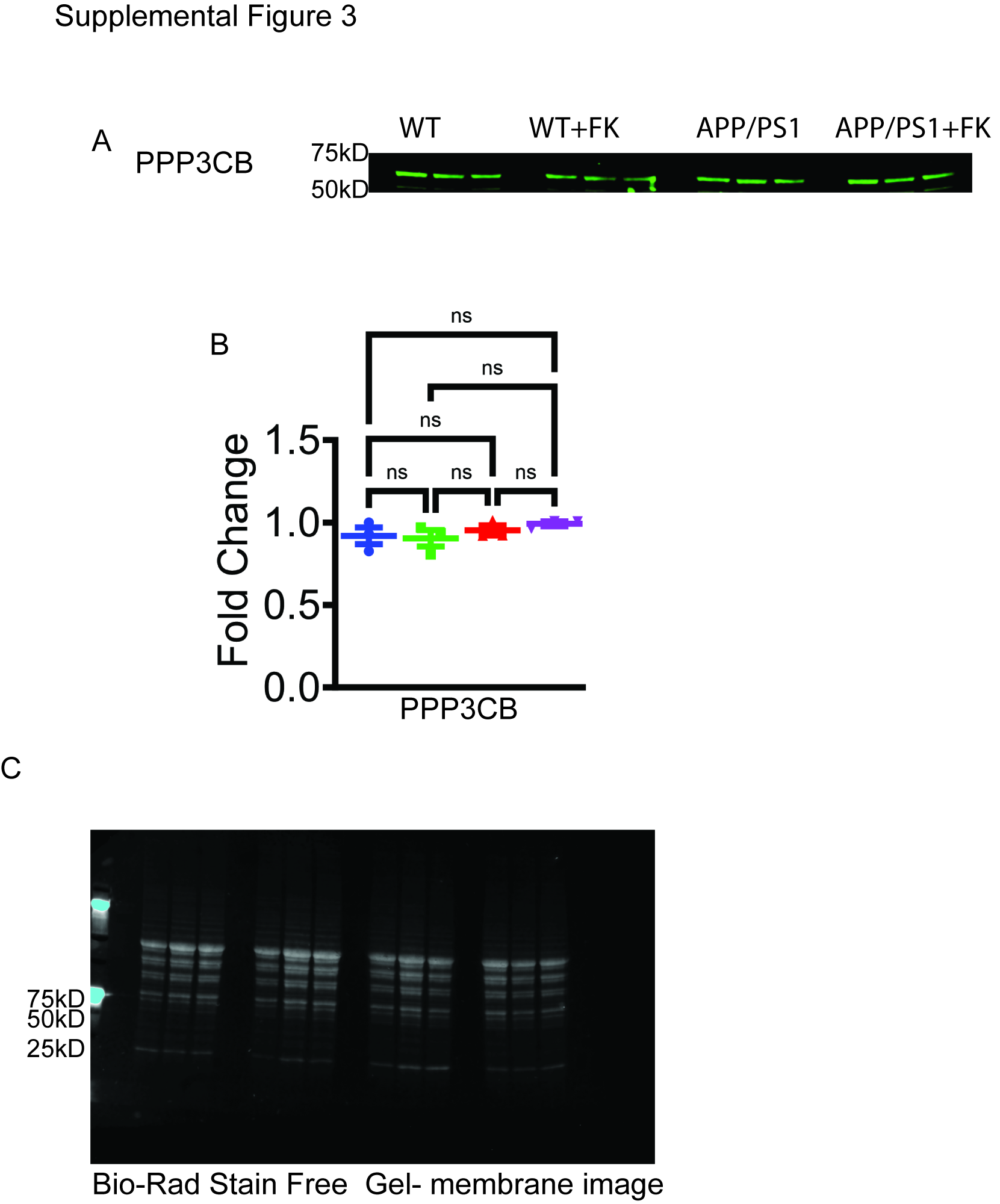

Supplement: Supplementary file 3 — Additional file 3: Supplemental Figure 3. CN levels in the cortex. (A) Immunoblot of PPP3CB from cortical lysates. (B) Fold change in PPP3CB levels compared to WT mice. n=3 mice per group. * = p < 0.05 by one-way ANOVA with Tukey. (C) Total protein loaded on blots from a Bio-Rad stain free gel and used for quantification in (B). For all graphs, WT (blue), WT+FK506 (green), APP/PS1 (red), and APP/PS1+FK506 (purple). [file 13195_2023_1323_MOESM3_ESM.tif]

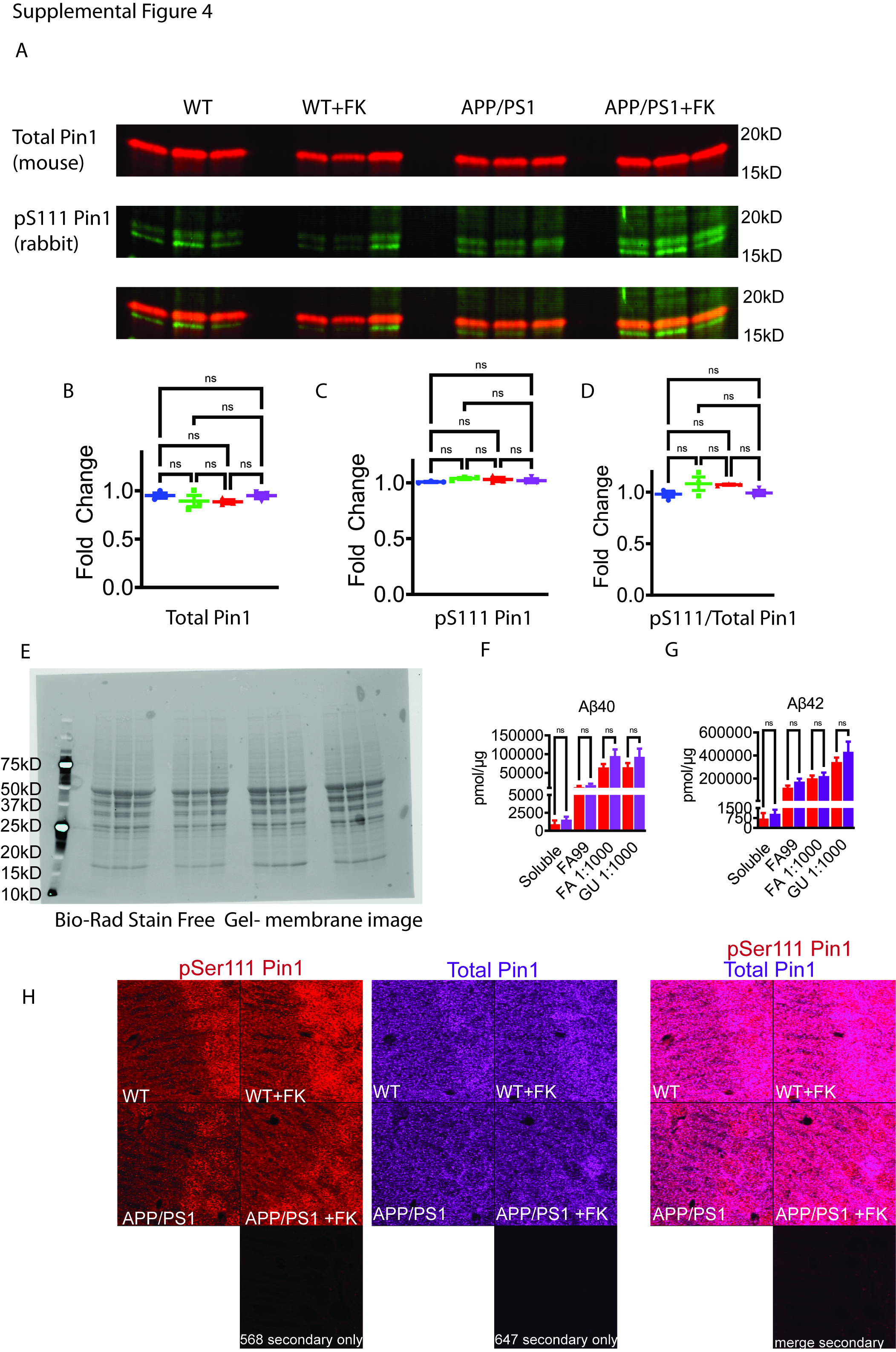

Supplement: Supplementary file 4 — Additional file 4: Supplemental Figure 4. Cortical Pin1 expression and phosphorylation and Aβ40/42 levels. (A) Western blot of Pin1 from cortical lysates using anti-total and anti-pSer111 Pin1 antibodies. (B) Quantification of total Pin1 protein levels from (A) n=3 mice per group. (C) Quantification of pSer111 Pin1 protein levels from (A). n=3 mice per group. (D) Ratio of fold change of pS111/total Pin1 in Western blots. n=3 mice per group. (E) Total protein loaded on blots from a Bio-Rad stain free gel and used for quantification in (B-D). Mesoscale multiplex measurements of Aβ40 and Aβ42. (F) Aβ40 measurements from cortical lysates. n=3 mice per group. (G) Aβ42 measurements from cortical lysates. n=3 mice per group. (H) Images from Fig. 2 with secondary only controls. All images adjusted to same intensity. For all graphs, WT (blue), WT+FK506 (green), APP/PS1 (red), and APP/PS1+FK506 (purple). [file 13195_2023_1323_MOESM4_ESM.tif]
